# Phenotypic Plasticity of Fibroblasts during Mammary Carcinoma Development

**DOI:** 10.3390/ijms20184438

**Published:** 2019-09-09

**Authors:** Eiman Elwakeel, Mirko Brüggemann, Annika F. Fink, Marcel H. Schulz, Tobias Schmid, Rajkumar Savai, Bernhard Brüne, Kathi Zarnack, Andreas Weigert

**Affiliations:** 1 Institute of Biochemistry I, Faculty of Medicine, Goethe-University Frankfurt, 60590 Frankfurt, Germany; elwakeel@biochem.uni-frankfurt.de (E.E.); t.schmid@biochem.uni-frankfurt.de (T.S.); b.bruene@biochem.uni-frankfurt.de (B.B.); 2 Buchmann Institute for Molecular Life Sciences (BMLS), Goethe University Frankfurt, 60438 Frankfurt, Germany; mirko.brueggemann@bmls.de; 3 Institute of Cardiovascular Regeneration, Faculty of Medicine, Goethe-University Frankfurt, 60590 Frankfurt, Germany; marcel.schulz@em.uni-frankfurt.de; 4 Max Planck Institute for Heart and Lung Research, Member of the German Center for Lung Research (DZL), Member of the Cardio-Pulmonary Institute (CPI), 61231 Bad Nauheim, Germany; Rajkumar.Savai@mpi-bn.mpg.de; 5 Frankfurt Cancer Institute, Goethe-University Frankfurt, 60596 Frankfurt, Germany; 6 Project Group Translational Medicine and Pharmacology TMP, Fraunhofer Institute for Molecular Biology and Applied Ecology, IME, 60590 Frankfurt, Germany; 7 German Cancer Consortium (DKTK), Partner Site Frankfurt, 60590 Frankfurt, Germany

**Keywords:** cancer-associated fibroblasts, mammary carcinoma, cancer, transcriptional profiling, gene signature

## Abstract

Cancer-associated fibroblasts (CAFs) in the tumor microenvironment contribute to all stages of tumorigenesis and are usually considered to be tumor-promoting cells. CAFs show a remarkable degree of heterogeneity, which is attributed to developmental origin or to local environmental niches, resulting in distinct CAF subsets within individual tumors. While CAF heterogeneity is frequently investigated in late-stage tumors, data on longitudinal CAF development in tumors are lacking. To this end, we used the transgenic polyoma middle T oncogene-induced mouse mammary carcinoma model and performed whole transcriptome analysis in FACS-sorted fibroblasts from early- and late-stage tumors. We observed a shift in fibroblast populations over time towards a subset previously shown to negatively correlate with patient survival, which was confirmed by multispectral immunofluorescence analysis. Moreover, we identified a transcriptomic signature distinguishing CAFs from early- and late-stage tumors. Importantly, the signature of early-stage CAFs correlated well with tumor stage and survival in human mammary carcinoma patients. A random forest analysis suggested predictive value of the complete set of differentially expressed genes between early- and late-stage CAFs on bulk tumor patient samples, supporting the clinical relevance of our findings. In conclusion, our data show transcriptome alterations in CAFs during tumorigenesis in the mammary gland, which suggest that CAFs are educated by the tumor over time to promote tumor development. Moreover, we show that murine CAF gene signatures can harbor predictive value for human cancer.

## 1. Introduction

Fibroblasts are the main cellular component of connective tissue. They are defined as spindle-shaped cells that shape the extracellular matrix (ECM) by producing its major building blocks such as collagens, fibronectins, and proteoglycans and by fine-tuning their arrangement through proteases such as matrix metalloproteinases (MMPs) [1]. A molecular definition of fibroblasts is challenging, since they show a remarkable degree of heterogeneity resulting mainly from genetic imprinting in their local microenvironment across anatomical sites and their multiple cellular origins. Fibroblasts are derived from diverse embryonic sources and a variety of cells can transdifferentiate to fibroblasts once homeostasis is disturbed [2,3,4]. Molecular markers associated with fibroblasts such as vimentin, the platelet-derived growth factor receptor chain α (Pdgfra), CD90, and collagens are not expressed by all fibroblasts and are, moreover, also expressed by other cells including endothelial cells, perivascular smooth muscle cells, immune cells and myoepithelia [1,2,3]. Fibroblasts are usually quiescent, but are activated when homeostasis is disturbed, e.g., during tissue injury. Fibroblast activation is usually triggered in response to tissue injury by wound-enriched factors such as transforming growth factor β (TGF-β), but also by a number of cytokines, other growth factors such as PDGF, activators of Wnt/β-catenin signaling, Toll-like receptor ligands, and reactive oxygen species, among others [5,6,7]. Activated fibroblasts, also called myofibroblasts, show an enhanced proliferative potential, synthesize increased amounts of ECM proteins, and aid in ECM remodeling. In addition, they acquire activation markers such as α smooth muscle actin (αSMA), which enables them to actively contract wound edges [1]. In wounds, fibroblast activation is reversible due to apoptotic death and repopulation with non-activated fibroblasts [4,8]. However, if the tissue injury stimulus persists, the healing response continues unabated. Thus, unrestricted fibroblast activation results in fibrosis characterized by an excessive accumulation of ECM. Fibrosis may destroy normal tissue architecture and consequently provoke loss of organ function [4,9].

Activated fibroblasts are important cellular players in the development of not only fibrosis, but also cancer. In this pathological condition, the desmoplastic reaction triggered by chronically activated fibroblasts, termed cancer-associated fibroblasts (CAFs), is one of the reasons why a tumor is considered “a wound that does not heal” [10]. CAFs share similarities with myofibroblasts in wounds, particularly the expression of αSMA, increased ECM synthesis, and enhanced ECM remodeling. They are generated by similar factors including TGF-β and PDGF, which also induce CAF proliferation and expansion [4]. However, a key distinction between wound-associated fibroblasts and CAFs is epigenetic programming of CAFs that renders them resistant to cell death, and maintains them in an activated state [11,12]. In this state, CAFs are usually linked to promoting tumor development by supporting tumor growth, invasiveness, and epithelial-to-mesenchymal transition (EMT), as well as by suppressing anti-tumor immunity [13,14,15,16,17,18,19,20]. On the contrary, in pancreatic ductal adenocarcinoma (PDAC), the presence of CAFs was linked to improved immune control and the production of tumor-restraining rather than tumor-supporting ECM [21,22]. These findings point to a heterogeneity of CAF phenotypes in tumors. Indeed, it has been noted that CAFs can derive from a diverse set of immediate progenitors, depending on tumor entity and experimental model, including resident fibroblasts, mesenchymal stem cells, pericytes, pre-adipocytes, and myeloid progenitors [4,23]. For instance, two spatially separated and functionally different subtypes of CAFs were identified in PDAC [17]. Similarly, two CAF subtypes exist in oral squamous cell carcinoma, which were suggested as two different developmental stages of CAFs [24]. With respect to mammary carcinoma, a recent study in the transgenic polyoma middle T oncogene (PyMT)-induced mammary carcinoma mouse model described four subtypes of CAFs by single-cell RNA sequencing (RNA-seq) [25]. The subtypes were suggested to be derived from distinct cellular sources. Whereas matrix CAFs (mCAFs) and cycling CAFs (cCAFs) originate from resident fibroblasts, vascular CAFs (vCAFs) shared endothelial markers and were suggested to originate from a perivascular location. Developmental CAFs (dCAFs) corresponded to malignant cells having undergone an EMT. Importantly, a transcriptomic signature corresponding to vCAFs was associated with poor prognosis and metastasis in mammary cancer patients [25]. Thus, CAF heterogeneity may be relevant from a therapeutic point of view. Besides these reports that clearly indicate CAF heterogeneity in a single tumor at a given time point, the development of CAF subsets over time is largely unexplored.

Here, we investigated the nature of fibroblasts in the untransformed mammary gland and in the PyMT mouse model at early- and late-stage carcinoma. Combining multispectral immunofluorescence with transcriptional profiling, we determined the plasticity of CAFs over time. Importantly, the accompanying changes in gene expression are linked to tumor stage and survival in human breast cancer patients, underlining the pathological relevance of the observed changes. 

## 2. Results 

### 2.1. Varying Fibroblast Subtypes during Mammary Gland Transformation and Tumor Development 

**Figure 1:**
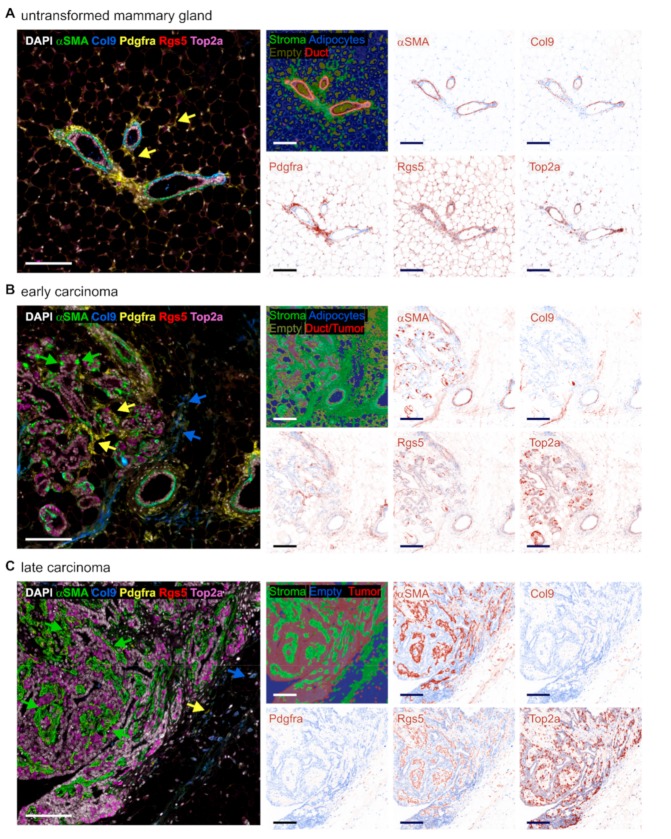
Histology of cancer-associated fibroblast (CAF) subset marker expression during PyMT tumor development. Untransformed mammary glands (**A**) as well as early (8–12 weeks) (**B**) and late-stage (18–20 weeks) (**C**) PyMT tumors were harvested and CAF subset marker expression was analyzed by PhenOptics. Representative images show expression of the activated fibroblast marker αSMA, the developmental CAF (dCAF) marker Col9, the matrix CAF (mCAF) marker Pdgfra, the vascular CAF (vCAF) marker Rgs5, and the cycling CAF (cCAF) marker Top2a. Nuclei were counterstained with DAPI. Large images display coexpression of all markers, with colored arrowheads indicating fibroblasts expressing the marker shown in the same color. The smaller images show tissue segmentation and single stainings computed using InForm software. Scale bars: 100 µm.

To analyze the heterogeneity of the CAF population in developing mammary tumors, we first investigated the abundance of different CAF subtypes in the untransformed mammary gland of 12-week-old mice, compared to early-stage tumors (8–12 weeks) and late-stage tumors (18–20 weeks) of mice expressing the PyMT oncogene in the mammary epithelium [26] (Figure 1A–C). Mammary glands with early-stage tumors usually contained hyperplasia or adenoma/mammary intraepithelial neoplasia (MIN), and rarely early carcinoma (comparable to human ductal carcinoma in situ with early invasion (DCIS + EI)), as defined by Lin et al. [26]. Mammary glands with late-stage tumors (18–20-week-old mice) also showed lesions in these stages, but all contained mainly tumors at the late carcinoma stage (which is comparable to human invasive ductal carcinoma in the PyMT model) [26]. Stromal cells were identified in the tissue sections by PhenOptics using the tissue segmentation algorithm in the InForm software that capitalizes both on autofluorescence and specific markers. We first aimed to discriminate four CAF subtypes previously described by Bartoschek et al. [25] and to track their abundance during the development of mammary carcinoma. We co-stained the prototypical CAF marker αSMA with an individual marker of each subtype, i.e., Rgs5 for vCAFs, Pdgfra for mCAFs, Top2a for cCAFs, and Col9 for dCAFs [25]. In the untransformed mammary gland, αSMA was only expressed by myoepithelial cells lining the epithelial layer of the mammary ducts (Figure 1A). Furthermore, these cells co-expressed Col9 and Rgs5, but not Pdgfra. In contrast, Pdgfra was expressed by cells lining the mammary ducts beyond the myoepithelial layer, and by cells interspersed into the adipocyte population, both corresponding to the resident mammary gland fibroblasts. Adipocytes expressed the vCAF marker Rgs5. The fibroblast phenotypes markedly diversified in the early-stage tumors of the transformed mammary gland. The myoepithelial layer around the transformed mammary ducts dissolved, giving rise to a mixed cell population that expressed αSMA, but rarely co-expressed Rgs5 and Col9. Pdgfra+ fibroblasts were still present, but a new subset of fibroblasts expressing mainly Col9, with low levels of Rgs5, emerged (Figure 1B). In late-stage tumors, the picture changed again. The dominating subset of fibroblasts within the tumor stroma were cells co-expressing αSMA and Rgs5, whereas cells expressing Pdgfra and Col9 were located extratumoral and were comparatively rare (Figure 1C). Throughout the tissues, the cCAF marker Top2a was mostly expressed by epithelial cells, whereas Top2a+ CAFs were rarely found. 

**Figure 2:**
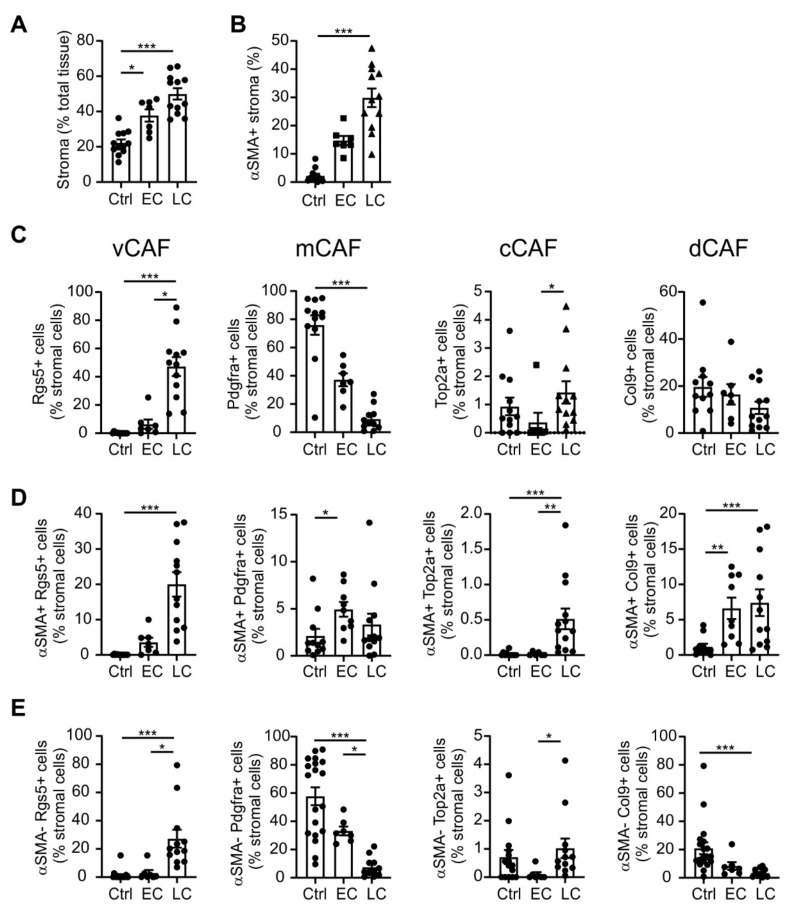
Quantitative analysis of cancer-associated fibroblast (CAF) subset marker expression during PyMT tumor development. Untransformed mammary glands (Ctrl) as well as early (EC; 8–12 weeks) and late-stage (LC; 18–20 weeks) PyMT tumors were harvested. CAF subset marker expression (αSMA, activated fibroblasts; Rgs5, vascular CAF (vCAF); Pdgfra, matrix CAF (mCAF); Top2a, cycling CAF (cCAF); Col9, developmental CAF (dCAF)) and tissue category abundance were analyzed by histology as in Figure 1. The abundance of stroma (**A**) and total αSMA+ stromal cells (**B**), as well as expression of CAF subset markers in total stroma (**C**), αSMA+ stromal cells (**D**) and αSMA− stromal cells (**E**). Individual data points indicate mean expression of markers in tissue sections of one individual animal (twelve individual animals were analyzed in the Ctrl and LC groups, and seven in the EC group). Additionally, means ± SEM are shown. *p*-values were calculated using one-way ANOVA with Bonferroni’s correction, * *p* < 0.05, ** *p* < 0.01, *** *p* < 0.001.

To monitor the extent of CAF plasticity during tumor development, we quantified the CAF subtypes in tissue sections of mammary tumors from different mice. The quantitative analysis confirmed a total increase in stroma during tumorigenesis over time, with a pronounced accumulation of αSMA+ cells in late-stage carcinoma (Figure 2A,B). At the level of total stroma, we found that cCAFs were barely detected, and dCAFs levels were not significantly altered (Figure 2C). Notably, vCAFs showed a slight increase in the early stage, and then massively rose in the late-stage carcinoma, when they dominated the CAF population (47% of all stromal cells). The sharp vCAF increase in late-stage carcinoma was apparent for both αSMA+ and αSMA− cells, suggesting a widespread accumulation of vCAFs in these tumors (Figure 2D,E). Accordingly, fibroblasts expressing mCAF marker Pdgfra, which had been most prominent in the untransformed mammary gland, progressively declined during tumorigenesis (Figure 2C). The mCAF decrease occurred at the level of quiescent cells (αSMA−, Figure 2E), whereas cells co-expressing αSMA and Pdgfra showed a mild but significant increase in early-stage tumors compared to the untransformed mammary gland cells (Figure 2D). While levels of Top2a+ stromal cells were still rare, Top2a+ αSMA+ cells significantly increased in late-stage tumors (Figure 2D). Despite the still low abundance of proliferating CAFs in late-stage tumors, this relative increase may suggest the appearance of proliferating cCAFs as the underlying reason for increased αSMA+ fibroblast numbers at this stage. The amount of dCAFs (αSMA+ Col9+ stromal cells) increased in tumors independent of stage, whereas αSMA− Col9+ cells were decreased in late-stage tumors. Overall, these data suggest a global shift in the CAF population during tumor progression. Already at the early stage, tumors show an altered CAF composition, which cumulates in a predominance of vCAFs in late-stage tumors, while the resident mCAF levels progressively retreat, at least at the level of non-activated fibroblasts.

### 2.2. A Gene Signature Separates Early versus Late-stage CAFs 

**Figure 3:**
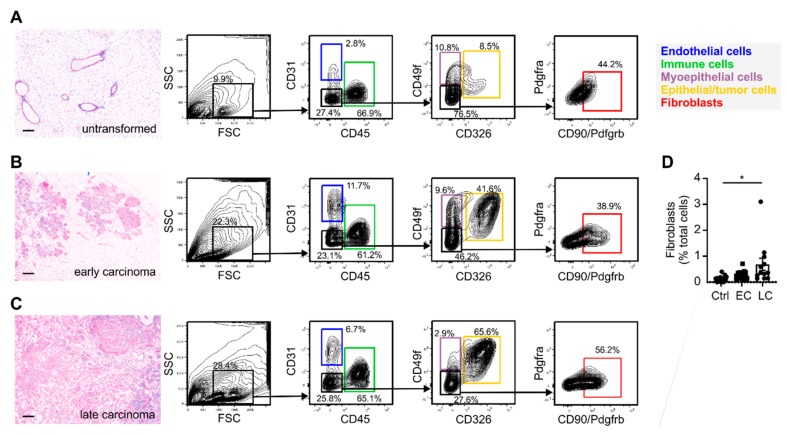
FACS of fibroblasts from untransformed mammary gland, early and late PyMT tumors. Untransformed mammary glands (Ctrl) as well as early (EC; 8–12 weeks) and late stage (LC; 18–20 weeks) PyMT tumors were harvested. Single cell suspensions were stained for the markers indicated and subjected to flow cytometric analysis and FACS-Sorting. Fibroblasts were identified as CD31− CD45− CD49f− CD326− Pdgfrb+ cells. Mock H&E images (scale bars: 100 µm) indicating tissue architecture and the sorting strategies for untransformed mammary glands (**A**), early- (**B**) and late-stage (**C**) PyMT tumors and the quantification of fibroblast abundance (**D**) are displayed. (**D**) Individual data points, means + SEM are shown. *p*-values were calculated using one-way ANOVA with Bonferroni’s correction, * *p* < 0.05.

**Figure 4:**
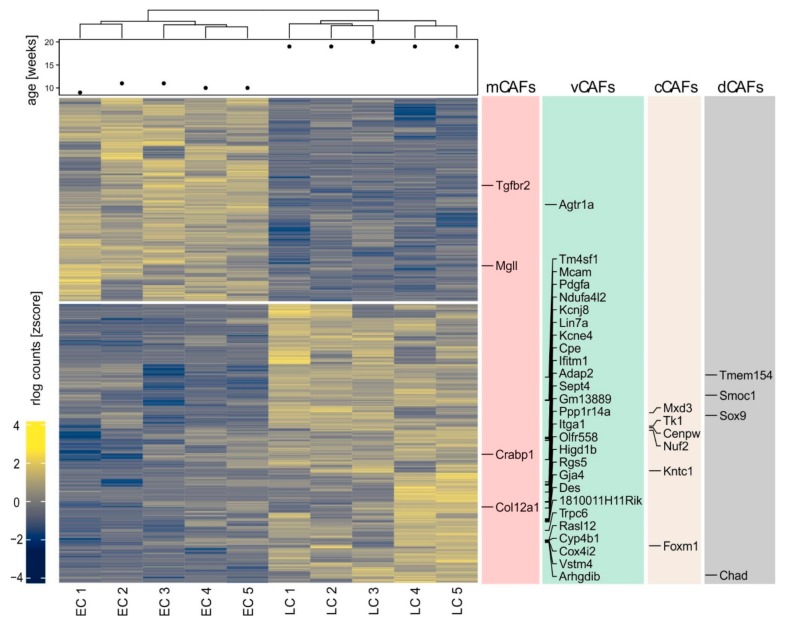
Comparative transcriptome analysis of early- and late-stage PyMT cancer-associated fibroblasts (CAFs). Transcriptomes of FACS-sorted early (EC) and late-stage (LC) PyMT CAFs were generated by mRNA sequencing. The heat map shows differentially expressed genes between both groups. Genes corresponding to individual CAF subsets (matrix CAFs, mCAFs; cycling CAFs, cCAFs; vascular CAFs, vCAFs; and developmental CAFs, dCAFs) are highlighted.

To investigate the changes in the CAFs between tumor stages in more detail, we FACS-sorted fibroblasts from the untransformed mammary gland, and early- and late-stage PyMT tumors [26] (Figure 3A–C). Fibroblasts were identified as cells lacking expression of the endothelial cell marker CD31, the immune cell marker CD45, and the epithelial markers CD326/Epcam and CD49f/Itga6, but expressing CD140b/Pdgfrb and/or CD140a/Pdgfra. CD49f marks myoepithelial cells that co-express fibroblast markers such as αSMA, Col9 and Rgs5 (Figure 1A). It was therefore essential to exclude these cells to obtain a pure fibroblast population. Control samples were analyzed to obtain a baseline setting from which tumor development could be followed. Using this baseline, we noticed, as expected, a relative increase in epithelial cells in mammary glands of PyMT mice over time. Additionally, we observed an increased abundance of fibroblasts, particularly in late-stage carcinoma (Figure 3D), confirming the histological observations (Figure 2A,B) at another quantitative level. After FACS-sorting (CAFs from tumors of five individual animals per stage), the transcriptome of the isolated fibroblasts from early- and late-stage tumors was determined by mRNA sequencing (75-nt single end sequencing; ~50 M reads per sample). To identify genes that would discriminate early- and late-stage CAFs, we performed differential gene expression analysis using DESeq2 [27]. Since initial quality controls indicated batch effects and inter-animal variability, we implemented a series of corrections to detect expression changes explicitly caused by the tumor progression (Figure S1). This procedure identified 906 genes that displayed a significant differential expression in the CAFs from early to late-stage carcinoma (523 up- and 383 downregulated, adjusted *p* value < 0.01; Figure 4 and Table S1). In line with our in situ results, upregulated genes included numerous markers that were identified as unique signature genes for vCAFs in the previous single-cell transcriptomics study [25], further supporting the predominance of vCAFs in the late-stage tumors (Figure 4). Moreover, we noticed preferred expression of a limited number of genes marking cCAFs and dCAF (Figure 4), the former also supported by our histology data (Figure 2D). 

Using this gene signature, we performed gene set enrichment analysis (GSEA) and analyzed enrichment of reactome pathways as well as gene ontology (GO) terms. GSEA revealed that only two curated gene sets from the Molecular Signatures Database were altered (normalized enrichment score ≥ 1.4, *p*-value ≤ 0.01, FDR *q*-value ≤ 0.25) in late-stage compared to early-stage CAFs (Table 1). 

**Figure 5:**
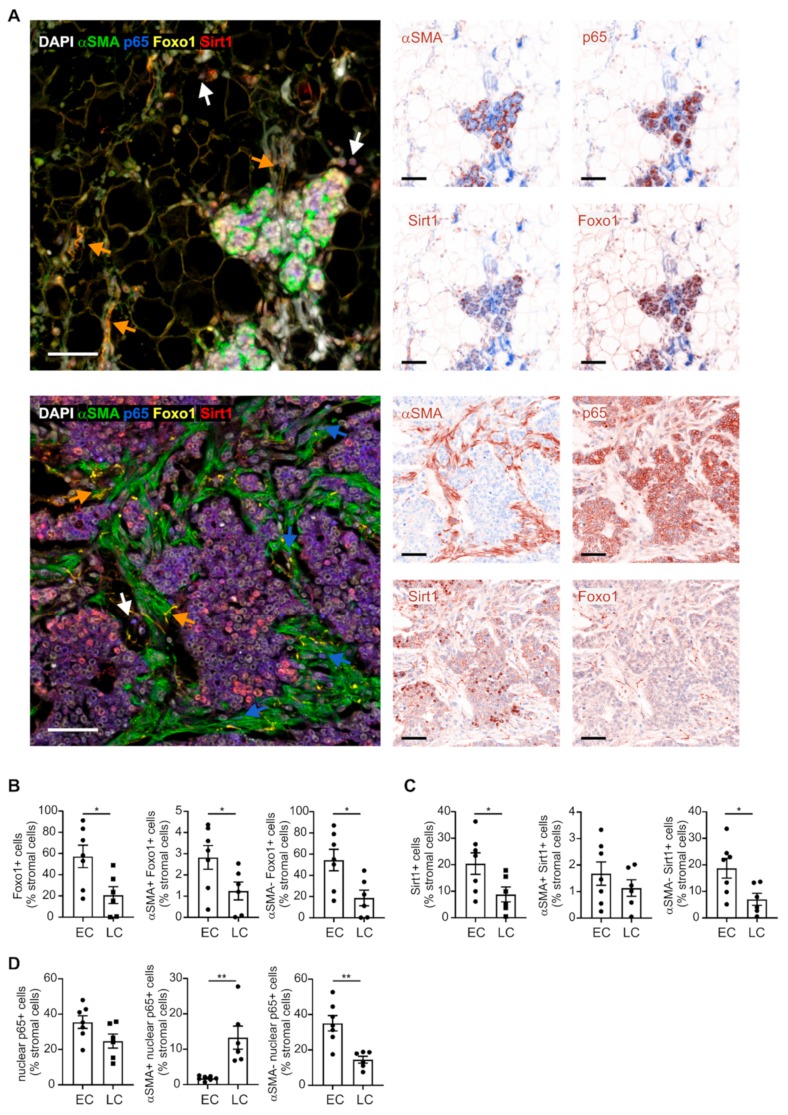
Histological validation of enriched gene signature in early versus late cancer-associated fibroblasts (CAFs). (**A**) Early- and late-stage PyMT tumors were harvested and analyzed for expression of nuclear p65, as well as Sirt1 and Foxo1 using PhenOptics. Nuclei were counterstained with DAPI. Representative images show combined expression of all markers as well as expression of single markers. Colored arrowheads indicate fibroblasts co-expressing Foxo1 and Sirt1 (orange) or α-SMA and nuclear p65 (blue), and nuclear p65-expressing lymphocytes (white). Scale bars: 50 µm. (**B**) Foxo1 expression in total stroma, αSMA+ stromal cells, and αSMA- stromal cells is displayed. (**C**) Sirt1 expression in total stroma, αSMA+ stromal cells, and αSMA− stromal cells is displayed. (**D**) Nuclear p65 expression in total stroma, αSMA+ stromal cells, and αSMA- stromal cells is displayed. Individual data points indicate mean expression of markers in tissue sections of one individual animal. Additionally, means ± SEM are shown. *p*-values were calculated using one-way ANOVA with Bonferroni’s correction, * *p* < 0.05, *** *p* < 0.01.

**Figure 6:**
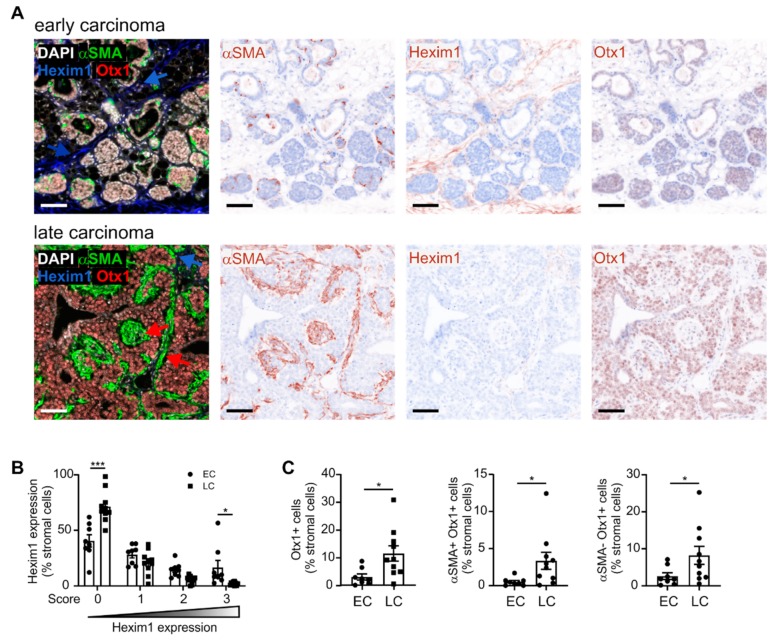
Histological validation of early versus late cancer-associated fibroblast (CAF) signature. (**A**) Early- and late-stage PyMT tumors were harvested and analyzed for expression of the activated fibroblast marker, as well as the predicted early CAF marker Hexim1 and the late CAF marker Otx1 using PhenOptics. Nuclei were counterstained with DAPI. Representative images show combined expression of all markers as well as expression of single markers. Scale bars: 50 µm. (**B**) Hexim1 expression in total stromal cells is shown. (**C**) Otx1 expression in total stroma, αSMA+ stromal cells, and αSMA- stromal cells is displayed. Individual data points indicate mean expression of markers in tissue sections of one individual animal. Additionally, means ± SEM are shown. *p*-values were calculated using one-way ANOVA with Bonferroni’s correction, * *p* < 0.05, *** *p* < 0.001.

These gene sets indicated an increased activity of the transcription factor nuclear factor kappa-light-chain-enhancer of activated B cells (NF-κB) in late-stage CAFs, corresponding to a number of cytokine and chemokine genes overexpressed in these cells when compared to early-stage CAFs (Table S1). Moreover, late-stage CAFs expressed a small number of genes upregulated in keratinocytes, particularly genes that were induced at high UV doses after 24 h [28]. These genes encompassed *Il1rn, Rela,* and *Cdc37*, again indicating increased inflammatory signaling in late-stage CAFs. Increased inflammatory signaling in late-stage CAFs was also apparent when looking at enriched reactome and GO terms (Table 1; shown are the most specific terms in a lineage), as indicated by terms related to immune cell chemotaxis, cytokine activity and inflammatory response. Besides, protease inhibitor activity, mainly attributable to the serine protease inhibitors (genes *Serpina1d, Serpina3f, Serpine2,* and *Serpini1*) as well as the MMP inhibitor *Timp1*, was also detected as a potentially enriched function of late-stage CAFs. When looking at early-stage CAFs, the only enriched terms were GO terms related to transcription (Table 1). This was due to an increase in the expression of transcription factors and other regulators of transcription including epigenetic regulators such as the histone acyltransferase *Ep300* and the histone deacetylase *Sirt1*. Interestingly, Sirt1 was previously connected to negative regulation of NF-κB signaling [29], and was shown to affect TGF-β signaling in fibroblasts [30]. Among the identified transcription factors, Foxo1 was connected to suppressing fibroblast proliferation, with the notion that inflammatory signaling suppresses Foxo1 transcription and activity [31,32]. We therefore tested the expression of Sirt1 and Foxo1 in CAFs in comparison with nuclear expression of the inflammatory NF-κB subunit p65, which is required for NF-κB signaling, by PhenOptics (Figure 5A). In both early- and late-stage tumors, we found cells with a fibroblast morphology co-expressing Foxo1 and Sirt1. However, these cells were significantly enriched in the stroma of early-stage tumors (Figure 5B,C). Nuclear p65 was also found in stroma of both early- and late-stage tumors. Nevertheless, only late-stage PyMT tumors contained fibroblasts expressing nuclear p65, with the notion that only αSMA-expressing but not Foxo1 and Sirt1-expressing fibroblasts showed nuclear p65. Stromal cells expressing nuclear p65 in early-stage tumor stroma had a lymphocyte morphology (Figure 5A). Quantitatively, we unexpectedly observed no difference in stromal cells expressing nuclear p65 between early- and late-stage tumors (Figure 5D). However, there was a strong increase in nuclear p65 in αSMA-expressing cells in late-stage tumors, while more αSMA-negative cells expressed p65 in early-stage tumors, which, again, were mainly lymphocytes (Figure 5D). These data indicate a reciprocal regulation of NF-κB signaling in different stromal cells during cancer development. Next, we tested a number of other antibodies against genes present in the signature of differentially expressed genes (DEGs) between early- and late-stage CAFs for determination of protein levels in PyMT tumors. Of those, antibodies targeting the proteins orthodenticle homeobox 1 (Otx1) and hexamethylene bisacetamide inducible protein 1 (Hexim1) were of sufficient quality for validation in PyMT carcinoma sections. Consistent with a decrease at the mRNA level, the Hexim1 protein was expressed at high levels in fibroblasts in the early stage, but its expression was low in late-stage carcinoma CAFs (Figure 6A,B). *Otx1* was increased at the mRNA level, reflected by a high expression of Oxt1 protein in late-stage carcinoma CAFs (Figure 6A,C), with the notion that also tumor cells expressed higher levels of Otx1 in the late stage (Figure 6C). Overall, histology data, thus, confirmed our findings at the transcriptome level. 

### 2.3. Changes in CAF Development in PyMT Tumors are reflected in Mammary Carcinoma Patients

**Figure 7:**
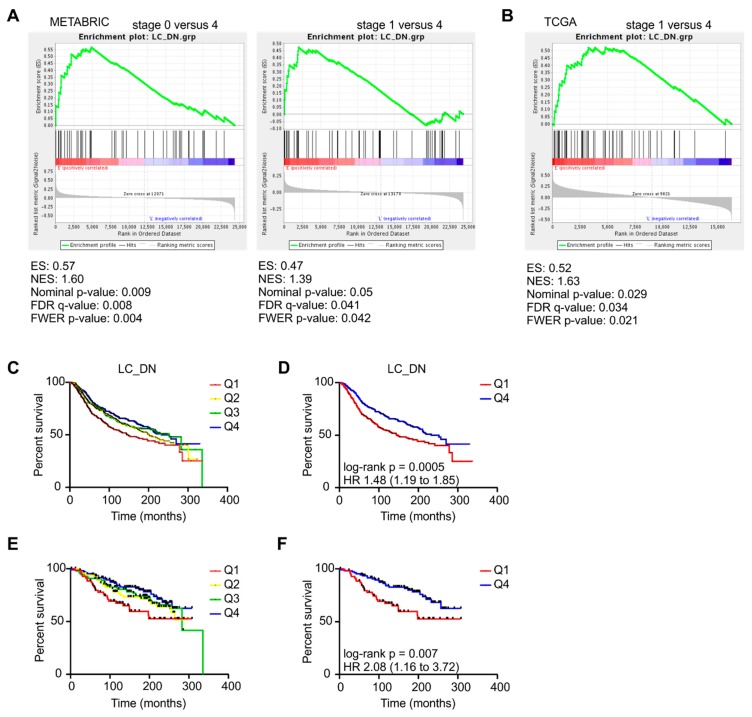
Correlation of an early PyMT CAF signature with human mammary carcinoma patient data. The METABRIC dataset and TCGA breast cancer dataset were analyzed for a correlation with an early PyMT CAF signature (= downregulated in late-stage CAFs (LC_DN), Table S2). Gene set enrichment analysis was performed to compare stage 0 and/or stage 1 mammary tumors with stage 4 mammary tumors in the METABRIC dataset (**A**) and TCGA dataset (**B**) using the early PyMT CAF signature as gene set input. (**C,D**) Patients were grouped into quartiles based on unranked mean expression of early PyMT CAF signature genes and survival rates were analyzed. Survival rates within all four quartiles (**C**) and of patients expressing high (>75% percentile) or lower (<75% percentile) levels early PyMT CAF signature genes (**D**). *p*-value was calculated using log-rank test. (**E,F**) Patients with stage 0 and stage 1 mammary tumors were grouped into quartiles based on unranked mean expression of early PyMT CAF signature genes and survival rates were analyzed. Survival rates within all four quartiles (**E**) and of patients expressing high (>75% percentile) or lower (<75% percentile) levels early PyMT CAF signature genes (**F**). *p*-value was calculated using log-rank test.

To investigate whether our findings in the murine PyMT model are relevant in human mammary carcinoma patients, we tested the expression of the genes discriminating early- versus late-stage CAFs in the published data from the Molecular Taxonomy of Breast Cancer International Consortium (METABRIC) and The Cancer Genome Atlas (TCGA). The METABRIC dataset contains gene expression and clinical data of ~2000 patients with mammary cancer [33], whereas TCGA dataset lists ~1000 invasive mammary carcinoma patients [34]. We performed a GSEA analysis with the human orthologs of genes that were upregulated in early- or late-stage PyMT CAFs, corresponding to 55 genes (PyMT early carcinoma (EC) signature), and 106 genes (PyMT late carcinoma (LC) signature), respectively (Table S2). When comparing stage 0 or stage 1 versus stage 4 human mammary carcinoma with our signatures using GSEA, we noticed a significant enrichment of the PyMT EC signature in early tumors of both datasets (FDR *q* value < 0.05; Figure 7A,B). Conversely, genes from the PyMT LC signature were enriched in late human mammary tumors, albeit it did not reach significance (data not shown). To test for prognostic capabilities of our gene signatures beyond tumor stage, we performed survival analyses with patient data from the METABRIC study. Notably, patients had a better survival prognosis when they expressed high levels of the genes that were predominantly expressed in early-stage CAFs (Figure 7C,D). This was at least partially independent of tumor stage as, even within stage 0/1 human mammary tumors alone, the gene signature of early-stage CAFs predicted favorable survival (Figure 7E,F). In line with the weak enrichment of the PyMT LC signature in human stage 4 mammary carcinoma, we did not find a correlation of this signature with patient survival (data not shown). These data indicate that early-stage CAFs are associated with increased survival of patients. 

**Figure 8:**
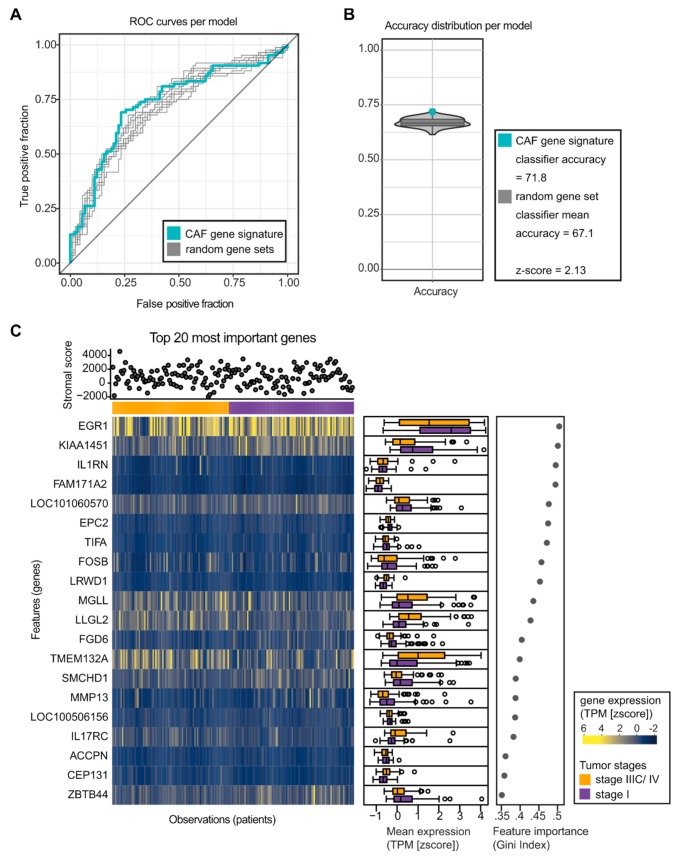
Random forest (RF) analysis to classify tumor stages in mammary carcinoma patients. A RF analysis was used to test the predictive power of the murine cancer-associated fibroblast (CAF) gene signature in staging of human breast cancer (patient cohort from TCGA dataset). (**A**) Receiver operating characteristic (ROC) curves based on the out-of-bag (OOB) error, for the RF trained on the 624 CAF signature genes (blue) and 10 RFs trained on random gene sets. (**B**) Accuracy distribution based on a 10-fold cross-validation for RFs trained on 100 randomly sample gene sets (grey), and the accuracy of the CAF signature gene set (blue). *z*-scores were obtained using the accuracy distribution from the 100 random gene sets. (**C**) Heat map that shows the influence of the 20 most informative genes in the RF classification (left). Genes are ordered by Gini index and patients are grouped according to their tumor stage (right). Gene expression is visualized as *z*-score transformed TPM values. The expression trend per tumor stage is captured for each gene in the form of boxplots (middle). Since the amount of stroma varies between samples, a stromal score is shown for each patient, which indicates the stromal content of each tumor tissue sample (top).

To further test if our murine CAF gene signature has predictive power in human breast cancer patients, utilizing information contained in both sets of DEGs (up- and downregulated), we performed random forest-based classification. The random forest model was trained on the entire CAF gene signature and compared to 100 randomly picked gene sets of comparable size. The CAF gene signature achieved an out-of-bag (OOB) error rate of 27% and consistently outperformed the random gene sets by 6% on average to predict tumor stage (Figure S2A). Accordingly, the receiver operating characteristic (ROC) curve showed a moderate dominance of the CAF gene signature-based model (AUC = 73%) over the random gene sets (Figure 8A). The predictions were largely independent of the fraction of stromal cells in the tumor samples [35], which showed no systematic differences between predicted and annotated tumor stages (Figure S2B,C). To minimize the risk of overfitting, we further validated the predictive power of our model by performing 10-fold cross-validation. The CAF gene signature-based model achieved an accuracy of 71.8%, which was superior to the distribution of accuracy values of the random gene sets (z-score = 2.13, Figure 8B). Random forest models provide an importance ranking of features with respect to their ability to correctly classify the test observations, measured as the decrease in classification accuracy upon permutation of the respective feature (Gini index). The top 20 genes according to the Gini index encoded, for instance, the transcriptional regulator EGR1, as well as components and products of inflammatory signaling pathways such as IL-1 (IL1RN, TIFA, MMP13) and IL-17 signaling (IL17RC), which are associated with fibroblast function [36,37,38,39] (Figure S2D). Moreover, EEF2K (encoded by the gene LOC10160570) limits fibroblast proliferation [40] and was accordingly among the genes downregulated in late-stage CAFs. Although the expression of the top 20 genes displays a certain level of variability between patients, a clear trend of regulation was captured for some of them (Figure 8C). Importantly, all of the top 20 genes in the CAF gene signature for which a reliable antibody staining was available were expressed in stromal cells in breast carcinoma section in the Human Protein Atlas [41]. EGR1, OSBPL8 (encoded by the gene KIAA1451), FAM171A2, FGD6, and CEP131 showed particularly high or largely exclusive staining in stromal cells, supporting the notion that indeed the expression profile of CAFs drives tumor classification in our random forest model. Together, these results underline that the information on gene expression changes in CAFs from our mouse experiments can be utilized to predict tumor stage in human breast cancer patients. Analyzing the CAF composition in the tumor microenvironment may therefore hold predictive value for human disease.

## 3. Discussion

The present study highlights once more that, as already described, the term CAF collectively refers to a heterogeneous group of cells. The PyMT model imposes some challenges on analyzing CAF phenotypes. Tumors develop in each of the ten mammary glands at different times. Moreover, within a single mammary gland, late- and early-stage tumors can be found at the same time [26]. When analyzing a single mammary gland in animals that are around 18–20 weeks old by histology (here defined as animals bearing late-stage tumors), we always observed tumors in the late carcinoma stage, but at the same time also hyperplastic, adenoma and/or early carcinoma lesions. In these early developmental stage lesions, fibroblast phenotypes were similar to those observed in mammary glands of younger mice (8–12 weeks) that do not yet contain late carcinomas. This feature likely leads to dilution of differences in CAF phenotypes when analyzing CAFs as a bulk population. Therefore, we likely observed only major differences in CAF phenotypes between early- and late-stage tumors (which we defined by the age of the mice), while subtle changes may have been missed. Moreover, this characteristic of the PyMT model suggests that rather the proximity to late-stage tumor cells or a corresponding microenvironment than the age of the mice per se is responsible for establishing late-stage fibroblast phenotypes. 

Cancer-associated fibroblasts are generally thought to support tumor growth, although their impact in different tumor stages has not been tested. Our data suggest that CAFs in the PyMT mammary carcinoma model are educated to a tumor-supportive phenotype over time, although they do not necessarily support tumor growth in early-stage carcinoma. Our signature of early-stage CAFs correlated well with early stage (stage 0 and/or 1) and favorable survival in human mammary carcinoma patients, suggesting an inhibitory impact of CAFs in early-stage breast tumors. This may sound counterintuitive, since accumulating evidence suggests that alterations in the ECM driven by activated fibroblasts precede tumor development. Fibrosis and a high mammographic density are risk factors for the development of breast cancer [42,43,44]. Moreover, mammary carcinomas are accompanied by sclerosis of the peritumoral extracellular matrix (ECM) [45,46]. Patients with germline BRCA1 mutations harbor dermal fibroblasts that show a CAF-like activation state and support rather than limit epithelial cell proliferation [47]. Nevertheless, these data only indicate that an altered ECM predisposes to the development of breast cancer. This still leaves room for an anti-tumoral role of recruited and/or converted CAFs during early progression of already established lesions, as suggested by our data. 

The signature of early-stage CAFs identified in our study predicted favorable survival of mammary carcinoma patients. However, gene expression datasets from complex tissues can only display gene expression in a mixed cell population. This is exemplified by Otx1, a transcription factor mainly expressed in neurons. Otx1 is also expressed in breast cancer cells, where it is thought to be induced by p53 to affect cancer stem cell differentiation [48]. We observed increased Otx1 expression in both CAFs and tumor cells. It is therefore unclear whether the prognostic relevance of OTX1 in human mammary carcinoma patients stems from its expression in stromal or cancer cells. 

While our signature of late-stage CAFs did not significantly correlate with stage in human mammary carcinoma and patient survival, we detected an enrichment of genes indicating the presence of vCAFs in late-stage PyMT tumors. A vCAF signature was previously associated with metastasis in human mammary carcinoma [25]. Since PyMT tumors start metastasizing to the lungs [26] within 18–20 weeks after birth in C57BL/6 PyMT mice, the increase of vCAFs in late-stage PyMT tumors appears rational. Future studies may selectively interfere with vCAFs generation or their functional program to test the impact of vCAFs on pulmonary metastasis. Concerning the latter, our late-stage CAF dataset was enriched in protease inhibitors, among them *Serpine2* and *Slpi*. These protease inhibitors were shown to contribute to metastasis by promoting vascular mimicry in breast cancer [49]. Therefore, they might be attractive targets to interfere with the pro-metastatic potential of vCAFs. 

Importantly, comparing our dataset with other published CAF datasets in different tumor entities (iCAFs, myCAFs, etc. [17,24]) using GSEA did not reveal enrichment of other published subtypes over time (data not shown). This may indicate that discrete CAF subtypes are formed dependent on the tissue origin of the tumor. 

The vCAF marker Rgs5 was prominently expressed by myoepithelial cells in the untransformed mammary gland, and to a lesser extent by adipocytes. The myoepithelial structure around the mammary ducts appeared to dissolve in early tumors, with few cells expressing both αSMA and Rgs5 around the tumor islands. When looking at the localization of the main CAF subset in late tumors, which still were localized between tumor islets, one may speculate that these αSMA and Rgs5 expressing cells might have stemmed from myoepithelial cells. Sophisticated fate mapping would be required to test this hypothesis. Recently, Raz et al. reported a decrease in Pdgfra-expressing fibroblasts in PyMT mammary tumors over time, which is confirmed by our data. They additionally observed an increase in bone marrow (BM)-derived CAFs that contributed substantially to the CAF pool in PyMT tumors [50]. We did not observe any markers indicating an increase in BM-derived cells (e.g., CD14, CD33, etc.) between early- and late-stage CAFs. This may be due to the fact that BM-derived CAFs accumulated already in tumors of 12-week-old mice, and were thus not specific for late-stage tumors. Moreover, BM-derived CAFs did not express high levels of αSMA and therefore are unlikely contributors to the dominant αSMA and Rgs5-expressing CAFs in late PyMT carcinomas.

GSEA, reactome and GO term analysis provided only little insight into the difference between early- and late-stage CAFs. The enriched gene sets provided a hint for increased inflammatory signaling in late-stage CAFs that may have been occurred via the transcription factor NF-κB. NF-κB indeed is a well-established driver especially of the inflammatory properties of CAFs that promote tumor growth [51]. Accordingly, we observed an increase in the NF-κB subunit p65 in the nucleus of αSMA-expressing late-stage CAFs, while nuclear p65 expression was higher in lymphocytic cells in early-stage tumors. It is important to note that NF-κB signaling in lymphocytes was mainly connected to their anti-tumor function, which is lost upon tumor development [52]. Interestingly, classical NF-κB target genes such as *Il6* and *Tnfa* were not expressed at higher levels in late-stage PyMT CAFs. Therefore, mechanisms that fine-tune the NF-κB response in CAFs need to be determined. Such analyses would need to include other levels of regulation of gene expression including mRNA stability, epigenetics and post-translational regulation of transcriptional regulators, which occur in CAFs, but were not addressed by the methodology employed in our study. It is unclear why NF-κB activity was higher in late-stage CAFs. Interestingly, both Foxo1 and Sirt1, which were highly expressed in early-stage CAFs and never co-expressed with nuclear p65, were connected to NF-κB signaling previously. Sirt1 is a direct negative regulator of NF-κB by deacetylating p65 [29]. Foxo1 was shown to synergize with NF-κB in the nucleus, but to be transcriptionally repressed by NF-κB [32]. The cytosolic localization of Foxo1 in early-stage CAFs and the absence of its expression in late-stage CAFs that show nuclear p65 support the pattern of increased NF-κB signaling in late-stage CAFs. Our data therefore indicate that activation of NF-κB signaling is a late event during CAF development at least in the PyMT model. 

In a study by Calvo et al. fibroblasts were isolated from PyMT tumors in different stages, cultured, immortalized, and then subjected to transcriptome analysis by microarray [53]. These analyses revealed that fibroblasts from hyperplastic tissue and adenomas rather than from carcinomas showed an NF-κB signature. However, the signature of these cells, which were expanded and immortalized is unlikely to be comparable with a signature obtained from freshly isolated CAFs. Calvo et al. rather observed an increased Yes-associated protein (Yap) signature in late-stage CAFs via GSEA. While we did not observe such a complete signature in our dataset, our late-stage CAF signature showed increased expression of Tead4, a main transcription factor through which Yap transmits its effects on gene expression [54]. 

Besides modulating inflammation, CAFs are often connected to modification of the ECM in tumors. When considering genes regulating the ECM, only *Col12a1* and three matrix metalloproteinases (*Mmp8*, *Mmp12*, and *Mmp13*) were upregulated in late-stage CAFs. *Col12a1* encodes collagen XII, which is a member of the FACIT collagens (fibril-associated collagens with interrupted triple helices). Collagen XII binds to collagen I-containing fibrils to connect them to associated matrix proteins such as decorin or tenascin, thereby forming flexible bridges between collagen I fibers [55]. Interestingly, *Col12a1* was previously connected to metastasis in breast and colon cancer [56]. Similarly, *Mmp13* has been connected to increased breast cancer metastasis [57]. In contrast, *Mmp8* and *Mmp12* have been ascribed a protective role in cancer, although this may be independent of their ability to shape the ECM [58]. Activity of MMPs is difficult to judge based on our data since we only tested mRNA levels of these factors. Moreover, our dataset may also suggest a rather negative impact on MMP activity based on the expression of the MMP inhibitor Timp1 in late-stage CAFs. Therefore, the association of CAF development with ECM modulation needs to be tested independently using other methods. 

We predicted stages in human mammary carcinoma based on our 624 CAF gene signature, which combines both up- and downregulated genes, using random forest analysis. Random forest models are commonly used for such tasks in biomedical research due to their versatility for large-scale datasets, while achieving a high accuracy. The resulting model indicates that the CAF gene signature is a suitable predictor for mammary carcinoma stage in humans. Despite having only an accuracy of 71.8%, the classifier trained with the CAF gene signature performed consistently better than a classifier trained with a random gene set. This underpins the predictive capacity of the identified gene set. However, when we investigated the expression of the most important genes that contributed to our model, we observed a relatively noisy expression pattern that reflect the rather high error rate. However, some of the genes show a visible expression trend per tumor stage, indicating the regulatory changes in gene expression during tumor progression. Additionally, we found genes highly expressed in breast cancer stroma in the Human Protein Atlas (EGR1, OSBPL8, FAM171A2, FGD6, and CEP131), or likely expressed in fibroblasts (MMP13), that are reported to play a role in breast cancer progression among the most important ones [59,60,61,62,63]. Their specific role in mammary carcinoma CAFs remains to be determined. This finding hints towards a species-conserved gene signature, potentially relevant in diagnostics and clinical practice. 

In conclusion, besides generating hypotheses as outlined above, our data add predictive value to CAF phenotypes that change during breast cancer progression. While early-stage CAFs may restrict tumor growth, late-stage CAFs are associated with metastasis. Taken together, we demonstrate alterations in CAF phenotypes during mammary tumor progression that are of relevance in human mammary carcinoma. Our data moreover provide new targets whose manipulation may allow switching CAF phenotypes, thereby potentially improving the outcome of mammary tumor development. 

## 4. Materials and Methods

### 4.1. Animal Experiments

Mice expressing the polyoma virus middle T oncoprotein (PyMT) under the Mouse Mammary Tumor Virus (MMTV) promoter in a C57BL/6 background were used. In the PyMT model, the expression of the PyMT oncoprotein is restricted to the mammary epithelium, which results in the appearance of mammary tumors starting from 6 weeks after birth in C57BL/6 mice and the occurrence of pulmonary metastases starting after 18 weeks [64]. For all animal experiments, the guidelines of the Hessian animal care and use committee were followed (approval number FU/1095, 12 October 2015).

### 4.2. Flow Cytometry 

For FACS-sorting of fibroblasts, tissue single suspensions were generated using the gentleMACS dissociator and the mouse Tumor dissociation kit (both from Miltenyi Biotec, Bergisch Gladbach, Germany). Single cell suspensions were stained with fluorochrome-conjugated antibodies and sorted using a FACS Aria III cell sorter (BD Biosciences, Heidelberg, Germany). Data were analyzed using FlowJo software Vx (BD Biosciences, Heidelberg, Germany). Antibodies and secondary reagents were titrated to determine optimal concentrations. CompBeads (BD Biosciences) were used for single-color compensation to create multi-color compensation matrices. For gating, fluorescence minus one (FMO) controls were used. The instrument calibration was controlled daily using Cytometer Setup and Tracking beads (BD Biosciences). The following antibodies were used: anti CD49f-PE-CF594, anti-CD140b-PE, anti-CD140a-APC, anti-CD326-BV711 (BD Biosciences), anti-CD31-PE-Cy7 (eBioscience, Frankfurt, Germany), and anti-CD45-VioBlue (Miltenyi Biotec). 7-AAD was used for dead cell exclusion.

### 4.3. cDNA Synthesis, Library Generation and Whole Transcriptome RNA Sequencing

RNA isolation and cDNA preparation of FACS-sorted fibroblasts were performed using the SMARTer^®^ Universal Low Input RNA Kit (Takara Bio, Saint-Germain-en-Laye, France) according to the manufacturer’s instructions. Prepared cDNA was purified by immobilization on AMPure XP beads (Beckman Coulter), and quantified using Qubit™ cDNA HS Assay Kits (ThermoFisher Scientific, Dreieich, Germany). Quality of purified cDNA was checked using the Agilent 2100 Bioanalyzer^®^ with High Sensitivity DNA Chips. Purified cDNA of sufficient quality was sheared using a M220 focused ultrasonicator (Covaris, Brighton, UK), yielding cDNA fragments around 400 bp. Fragmented cDNA was then used to prepare libraries using the SMARTer ThruPLEX DNA-Seq Kit (Takara Bio) according to the manufacturer’s instructions. Amplified libraries were purified by immobilization on AMPure XP beads, and quality and DNA content were checked using High Sensitivity DNA Chips as well as again with Qubit™ cDNA HS Assay Kits. Libraries were diluted, denatured according to Illumina Denture and Dilute Libraries Guide, and mixed with PhiX control (8%). Six to eight libraries were loaded on one sequencing cartridge of the TG NextSeq^®^ 500/550 High Output Kit v2 (75 cycles) (Illumina, Eindhoven, The Netherlands) and sequencing was performed on the NextSeq platform (Illumina).

### 4.4. RNA-seq Data Processing

Initial sequence quality was monitored with FastQC (V 0.11.5). Potential 3′ end degradation biases were visualized using PicardTools CollectRnaSeqMetrics (V 2.17.2). Using Flexbar (V 3.0.3) [65], adapter sequences were removed from the 3′ ends, and resulting reads were subjected to a window-based quality trimming (Phred score < 20, 5-nt window). Processed reads were mapped to the mouse genome (assembly GRCm38/mm10) based on GENCODE gene annotations (release m16) using STAR [66]. Reads were allowed to map with up to 5 mismatches, while considering no multi-mapping reads.

### 4.5. Differential Expression Analysis

The differential expression analysis was performed using the R/Bioconductor package DESeq2 (V 1.22.2) [27]. Read overlaps were counted within annotated exons using GenomicAlignments (V 1.18.1) in “union” mode. The resulting count matrix contained expression values for 53,379 mouse genes across 10 biological replicates (5 early-stage carcinoma, 5 late-stage carcinoma). Initial quality control via Principal Component Analysis (PCA) revealed a batch effect (Figure S1A,B). To account for this effect, changes in response to tumor stage were modeled using the design formula “design = ~date_batch + stage”. To detect expression changes explicitly caused by tumor progression and not by the batch effect, each variable was modeled separately (cooksCutoff = FALSE). Genes significantly regulated by tumor progression or batch were extracted by specifying the contrast argument (Figure S1C,D). Both sets were used to identify a subset of genes that are regulated by tumor progression, but unaffected by the batch (Figure S1E). This analysis yielded 906 differentially regulated genes, including 523 and 383 genes that were up- and downregulated in late-stage compared to early-stage carcinoma, respectively (adjusted *P* value < 0.1, Benjamini–Hochberg correction). k-means clustering with k = 2 separated genes in those that are up- or downregulated during tumor progression (Figure 4 and Figure S1F).

### 4.6. Analysis of Publicly Available Human Mammary Carcinoma Datasets

TCGA dataset and the METABRIC dataset [33] were downloaded from cBioPortal for Cancer Genomics (http://www.cbioportal.org) including associated clinical data. 

### 4.7. Assignment of Human-mouse Orthologs

TCGA dataset contained gene expression values for 20,531 genes (identified by Entrez IDs) in 1102 patients. First, genes were linked to stable Ensembl gene IDs [67] using the BioMart tool. Next, orthology assignments to putative mouse orthologs were extracted from the Orthologous MAtrix (OMA, release December 2018) [68]. This yielded 16,326 human genes in TCGA that had at least one ortholog in mouse, including 624 human orthologs out of the 906 differentially expressed mouse genes. We resolved 13 co-ortholog relationships by selecting a single representative.

### 4.8. GSEA, Reactome Pathway and GO Term Analysis

Differentially expressed gene between early- and late-stage carcinoma CAFs were used as an input to analyze gene sets in the Molecular Signatures Database [69] using GSEA 3.0 [70], as well as reactome pathways and GO terms using PANTHER V14 [71]. For correlation analysis between the PyMT EC or LC gene signatures and human mammary carcinoma datasets, gene lists of up- or downregulated genes in late-stage compared to early-stage carcinoma CAFs were generated using the following inclusion criteria: adjusted *P* value < 0.01, |log_2_ fold change in expression| > 1, and normalized base mean above 50. The lists were ranked based on adjusted *P* value. 

### 4.9. PhenOptics Immunofluorescence Analysis 

Opal^TM^ 7-Color Fluorescent Immunohistochemistry (IHC) Kits were used according to the manufacturer’s instructions. The following antibodies were used: αSMA (Sigma; F3777), Col9 SANTA CRUZ; sc-376969), Pdgfra (Cell Signaling; #3174), Rgs5 (Biozol; bs-2794R), Top2a (Biozol; orb379272), p65 (Cell Signaling; #8242), Foxo1 (Cell Signaling; #2880), Sirt1 (Upstate; 07-131), Hexim1 (Cell Signaling; #12604), and Otx1 (Abcam; ab25985). Stained tumor and mammary gland sections were scanned using Vectra^®^ 3 automated quantitative pathology imaging system and analyzed using InForm software V2.3. Expression of markers in cells was quantified using the Scoring algorithm of the InForm software, either 4 bin (Hexim1) or double positivity (all others markers) scoring. 

### 4.10. Random Forest Analysis 

The random forest analysis was implemented using the randomForest R package (version 4.6). Differentially expressed genes with an ortholog in TCGA dataset served as features for model training (*n* = 624). The model was trained for a binary classification task, to discriminate between early- and late-stage tumor patients. In total, the dataset comprised 174 patients of breast cancer stage I (*n* = 90) and stage IIIC/ IV (*n* = 84) according to the classification system by the American Joint Committee on Cancer (AJCC). A random forest consists of a collection of decision trees, where each tree is built from a random subset of features and observations, leading to robust classification results. The final classification result is the average across all trees in the forest [72]. The performance of the forest usually increases with the number of trees until it stabilizes. In our case, the error stabilized with 2000 trees (parameter *ntree*), while considering 19 gene features to be randomly sampled for each tree (parameter *mtry*). During model generation, an error rate is computed as the out-of-bag error. This is done by using all observations not used for the particular random training set, and using the left-out observations for estimation of the classification error [72]. Despite being very robust, the model might still overfit to the data [73]. Therefore, we implemented a 10-fold cross validation strategy, in which always 10% of the observations were left out, and the classification accuracy was estimated on this hold-out dataset. To assess the predictive power of our mouse-derived gene set, we compared the performance to 100 randomly sampled datasets of comparable size (*n* = 500). Random datasets were chosen from all human genes in TCGA dataset having an ortholog in mouse, while ignoring those genes already used in our main classifier (*n* = 15,702). For each gene set, a random forest classifier was trained with 2000 trees. Stromal scores for all samples in TCGA breast cancer dataset were obtained from the ESTIMATE webpage [35].

### 4.11. Statistical Analysis

Data were analyzed using GraphPad Prism 8.0 (GraphPad Software, San Diego, CA, USA).

*p*-values were calculated using two-tailed Student’s t-test, one-way or two-way ANOVA. To check for Gaussian distribution, D’Agostino and Pearson omnibus normality tests were performed. Differences in patient survival were analyzed using Log-rank (Mantel–Cox) test. Parametric or non-parametric tests were applied accordingly. Asterisks indicate significant differences between experimental groups (* *P* value < 0.05, ** *P* value < 0.01, *** *P* value < 0.001).

## Figures and Tables

**Table 1:** Gene sets, reactome and GO terms enriched in late-stage (LC) and early-stage EC) CAFs. ES, enrichment score; NES, normalized enrichment score; NOM, nominal; FDR, false discovery rate. +/− indicates enrichment or depletion of a given term.

Upregulated in LC				
**GSEA**				
**GENE SET NAME**	**ES**	**NES**	**NOM*****p***-**value**	**FDR q-val**
HINATA_NFKB_TARGETS_KERATINOCYTE_UP	0.79	1.81	<0.001	0.15
SESTO_RESPONSE_TO_UV_C0	0.78	1.78	<0.001	0.15
**Reactome**				
**Reactome pathways**	**Fold enrichment**	**+/−**	**Raw p-value**	**FDR**
Chemokine receptors bind chemokines	12.08	+	1.05E-04	2.41E-02
G alpha (i) signaling events	5.00	+	6.87E-07	1.10E-03
**GO**				
**PANTHER GO-Slim Biological Process**	**Fold enrichment**	**+/−**	**Raw p-value**	**FDR**
Granulocyte chemotaxis	14.49	+	8.35E-06	2.46E-03
Cytokine-mediated signaling pathway	8.45	+	3.45E-05	7.62E-03
Regulation of MAPK cascade	7.51	+	2.31E-04	2.55E-02
Inflammatory response	6.63	+	1.42E-04	1.80E-02
**PANTHER GO-Slim Molecular Function**	**Fold enrichment**	**+/−**	**Raw p-value**	**FDR**
Potassium channel regulator activity	19.10	+	8.92E-04	2.63E-02
Endopeptidase inhibitor activity	10.66	+	8.80E-06	4.90E-04
Cytokine receptor binding	9.87	+	1.93E-07	2.42E-05
Protease binding	9.25	+	2.03E-05	9.24E-04
Cytokine activity	8.76	+	5.27E-07	4.40E-05
G-protein coupled receptor binding	6.29	+	1.93E-04	6.90E-03
G-protein coupled receptor activity	3.45	+	2.78E-04	8.71E-03
**Upregulated in EC**				
**GO**				
**PANTHER GO-Slim Biological Process**	**Fold enrichment**	**+/−**	**Raw p-value**	**FDR**
Regulation of transcription by RNA polymerase II	3.97	+	9.12E-07	8.06E-04
Transcription by RNA polymerase II	3.10	+	2.84E-06	8.37E-04
**PANTHER GO-Slim Molecular Function**	**Fold enrichment**	**+/−**	**Raw p-value**	**FDR**
Transcription regulatory region DNA binding	4.55	+	1.88E-05	4.71E-03

## Supplementary Materials

The following are available online at https://www.mdpi.com/1422-0067/20/18/4438/s1.
