# Supplementary material for: Phenotypic Plasticity of Fibroblasts during Mammary Carcinoma Development
Source: Int J Mol Sci. 2019 Sep 9;20(18):4438. doi: 10.3390/ijms20184438 (PMC6769951; doi:10.3390/ijms20184438)
Supplement: Supplementary file 1 [file ijms-20-04438-s001.pdf]

Supplemental Figures and Figure Legends

Supplementary Figure 1

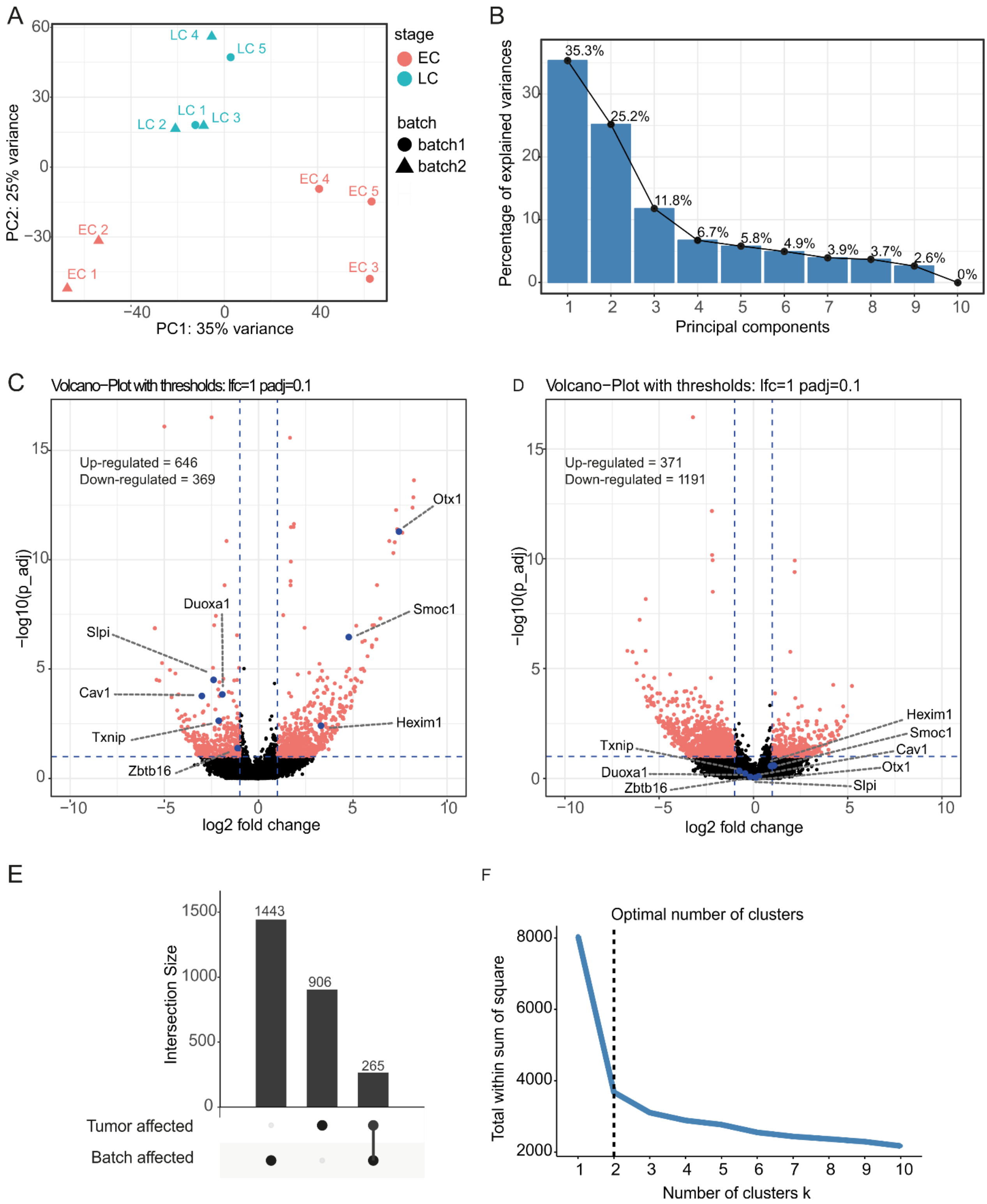

Figure S1. Differential expression analysis between early and late-stage carcinoma fibroblasts. Gene expression datasets of CAFs sorted from from 5 early-stage PyMT carcinomas and 5 late-stage PyMT carcinomas were generated in two

batches. (**A**) Principal component analysis (PCA) of all 10 replicates based on the 500 most expressed genes. Principal component (PC) 1 splits the samples by batch, component 2 splits samples by stage. (**B**) Variance distribution of all principal components calculated in the PCA. Components 1 and 2 describe more than 60% of the total variance in the dataset. (**C**) Volcano plot of all genes significantly regulated by the tumor stage (log2-fold-change threshold = 1, benjamini-hochberg corrected p-value threshold = 0.1). (**D**) Volcano plot of all genes significantly regulated by the batch (log2-fold-change threshold = 1, benjamini-hochberg corrected p-value threshold = 0.1). (**E**) Upset plot indicating the overlap between genes regulated by the tumor and the batch effect. (**F**) Ellbow plot for the k-means clustering of the 906 genes only regulated by the tumor stage. The sum of squared errors per k indicates that 2 clusters are sufficient.

Supplementary Figure 2

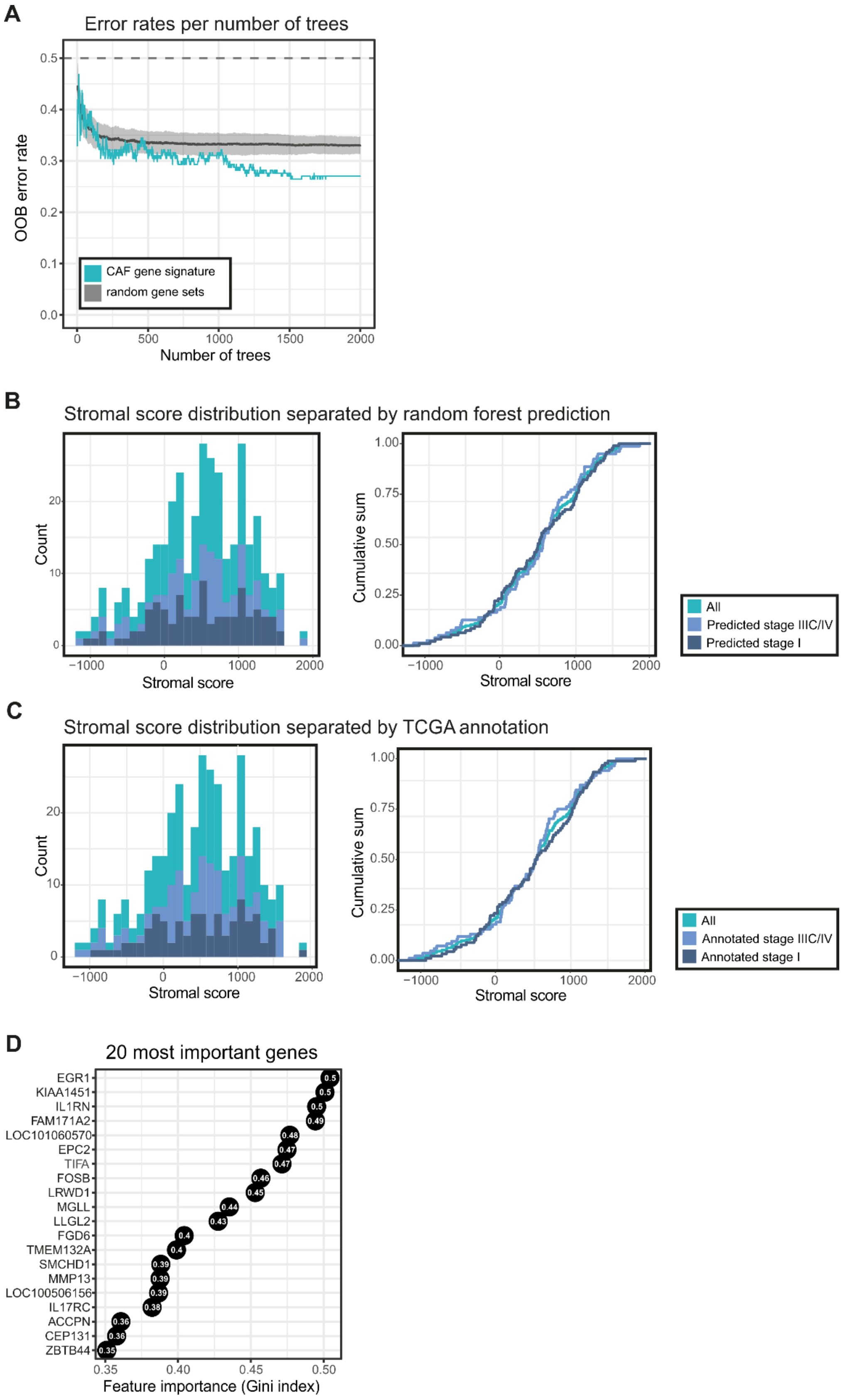

Figure S2. Features of random forest (RF) analysis to classify tumor stages in mammary carcinoma patients. A RF analysis was used to test the predictive power

of the murine CAF gene signature in staging of human breast cancer (patient cohort from the TCGA dataset). (**A**) Progression of the out-of-bag (OOB) error rate according to the number of trees grown in the RF. The OOB error rate for the RF classifier based on the CAF gene signatures (n=624) is shown in blue and the mean OOB for the RF classifiers based on 100 randomly sampled gene sets is shown in grey. (**B,C**) Stromal score distribution of all tumor tissue samples that were used for RF training compared to the predicted stage and the annotated stage. (**D**) 20 genes that contribute the most to the signature gene classifier RF.
